# Endometriosis in para-aortic lymph node resembling a malignancy: a case report and literature review

**DOI:** 10.1186/s12905-022-01659-4

**Published:** 2022-04-05

**Authors:** Jinjin Li, Yingwei Liu, Kaiwen Du, Lin Xiao, Xinyue He, Fengqin Dai, Junying Tang

**Affiliations:** 1 Department of Gynecology, The First Affiliated Hospital of Chongqing Medical University, No. 1 Youyi Road, Yuzhong District, Chongqing, 400016 People’s Republic of China; 2 Department of Pathology, The First Affiliated Hospital of Chongqing Medical University, No. 1 Youyi Road, Yuzhong District, Chongqing, 400016 People’s Republic of China

**Keywords:** Endometriosis, Para-aortic lymph node, Carbohydrate antigen 125, Case report

## Abstract

**Background:**

Endometriosis is a common benign gynecological disease characterized by growing-functioning endometrial tissue outside the uterus. Extra-pelvic endometriosis, which accounts for approximately 12% of endometriosis, is more challenging to diagnose because of its distance from the pelvic organs. Halban's theory of benign metastasis indicates that endometrial cells can appear in extra-pelvic organs via lymphatic and blood vessels, but endometrial lymph node metastasis cases are still rare. We report a case of endometriosis in a para-aortic lymph node whose clinical behavior mimicked a malignancy.

**Case presentation:**

A 52-year-old perimenopausal woman underwent laparoscopic hysterectomy plus bilateral salpingectomy (the patient insisted on the preservation of her ovaries) at a local hospital 2 years earlier because of adenomyosis. The patient presented with a complaint of low back pain to the gastrointestinal outpatient department of our hospital. The carbohydrate antigen 125 (CA125) was abnormally elevated at 5280.20 U/ml, human epididymis 4 (HE4) was 86.0 pmol/L, while other tumor markers were normal. Serum female hormone results were in the postmenopausal range, and her gastroenteroscopy showed no abnormalities. Moreover, both enhanced magnetic resonance imaging and positron emission tomography-computed tomography showed a high possibility of a retroperitoneal malignant lymph node (metastasis possible, primary site unknown). One week after admission, she underwent laparoscopic exploratory surgery, during which we observed normal shape and size of both ovaries while the left ovary was cystic-solid. After opening the retroperitoneal space, an enlarged lymph node-like tissue measuring 8 × 4 × 3 cm^3^ was found near the abdominal aorta. When the surrounding adhesions were separated, lymph node-like tissue was poorly demarcated from the abdominal aorta and renal artery. Some lymph node samples and left ovary were sent for intraoperative frozen section, which revealed benign lesions, similar to endometrial tissue. The lymph node tissue was then excised as much as possible, and the second set of intraoperative frozen sections showed high probability of endometrial tissue. The final histopathology and immunohistochemistry staining reached a diagnosis of para-aortic lymph node endometriosis. Gonadotropin-releasing hormone antigen treatment was recommended every 28 days because of the high preoperative CA125 and imaging-based suspicion of malignancy. The serum CA125 subsequently decreased to normal levels, and no para-aortic lesions were detected on abdominal enhancement CT. She is being followed up regularly.

**Conclusion:**

It is known that the incidence of lymph node metastasis in pelvic endometriosis is relatively rare. Our report shows that endometriotic tissue can metastasize via the lymphatic route and suggests that endometriotic tissue has the characteristics of invasion and metastasis.

## Background

Endometriosis (EMs) is defined as the presence of functional endometrial tissue (glands and stroma) outside the uterus. The clinical manifestations vary depending on the person and site of invasion, and symptoms are closely related to the menstrual cycle, including pelvic pain, dyspareunia, menstrual disorders, infertility, and even complete absence of symptoms [1, 2]. EMs mainly occurs in pelvic organs, especially the ovary and uterosacral ligament. Extra-pelvic EMs accounts for about 12% of EMs, and can occur in the gastrointestinal and urinary tracts, upper and lower respiratory system, diaphragm, thoracic cavity, pericardium, umbilicus, abdominal wall, vulva, brain, and large groups of muscles [3–5]. Extra-pelvic EMs are more challenging to diagnose because of their distance from the pelvic organs. Studies have reported that 84% of extra-pelvic EMs are diagnosed in patients with non-Gynecological symptoms [5].

Several studies on the pathogenesis of EMs have failed to come up with a definitive explanation, and although the theory of endometrial implantation has been widely accepted, it is challenging to explain extra-pelvic EMs based on this theory alone [5]. Halban's theory of benign metastasis indicates that endometrial cells can appear in extra-pelvic organs via lymphatic and blood vessels, but endometrial lymph node metastasis cases are still rare. We present the case of a 54-year-old woman who previously underwent hysterectomy but subsequently developed abnormally elevated CA125 levels and an enlarged para-aortic lymph node that was initially misdiagnosed as a retroperitoneal malignancy. However, she was eventually diagnosed with endometriosis in a para-aortic lymph node.

## Case presentation

A 52-year-old perimenopausal woman who suffered from progressive dysmenorrhea for 36 years was diagnosed with adenomyosis at a local hospital and underwent laparoscopic hysterectomy and bilateral salpingectomy (the patient insisted on the preservation of her ovaries) 2 years ago. She was not followed up regularly after the operation.

**Fig. 1:**
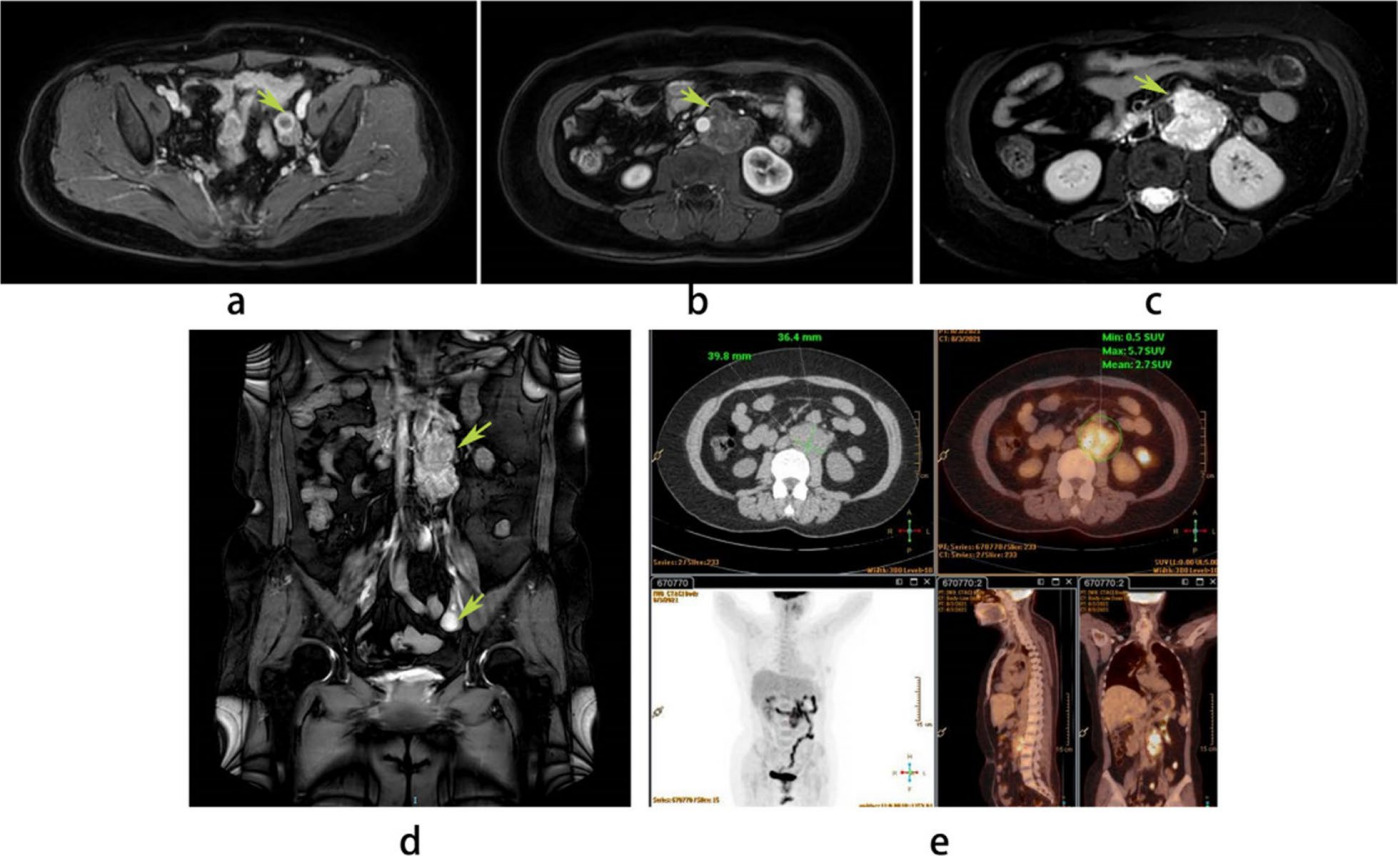
Findings on imaging of the patient. An MRI revealed that the left ovary was cystic and solid (**a**), and the irregular retroperitoneal mass at the lower margin of the first to the third lumbar spine measured approximately 75 × 42 × 37 mm^3^ (**b**–**d**). A PET-CT scan demonstrated that the lesion was on the left side of the retroperitoneal abdominal aorta about 70 × 40 × 36 mm^3^ in size with increased metabolic activity and unclear borders (**e**)

Three months ago, the patient attended the gastrointestinal outpatient department of our hospital with progressive aggravation of low back pain for more than one month. The serum CA125 was abnormally elevated (5280.20 U/ml, normal: 0–35 U/ml), HE4 was 86.0 pmol/L, carbohydrate antigen 199 and carcinoembryonic antigen levels were normal. Serum female hormones (anti Mullerian hormone, AMH: < 0.02 ng/ml; estradiol, E2: < 15 pg/ml; progestin, P:0.72 ng/ml; follicle-stimulating hormone, FSH: 27.31 mIU/ml; luteinizing hormone, LH: 8.03 mIU/ml) results suggested her postmenopausal status. Her gastroenteroscopy results showed no abnormality. Simultaneously, abdominal and pelvic enhanced magnetic resonance imaging (MRI) suggested that the left ovary was cystic and solid (Fig. 1a), and fluid or blood accumulation was considered. An irregular retroperitoneal mass at the lower margin of the first to the third lumbar spine measured approximately 75 × 42 × 37 mm^3^, which was considered to be a retroperitoneal malignant lymph node (Fig. 1b–d). After a multidisciplinary team (MDT) discussion by gastroenterologists, oncologists, radiologists, and gynecological oncologists, the patient was admitted to the gynecological department. Additionally, positron emission tomography and computed tomography (PET-CT) showed that the lesion was located on the left side of the retroperitoneal abdominal aorta, measuring about 70 × 40 × 36 mm^3^ in size with increased metabolic activity and unclear borders, with a high probability of being a malignant lymph node (Fig. 1e).

**Fig. 2:**
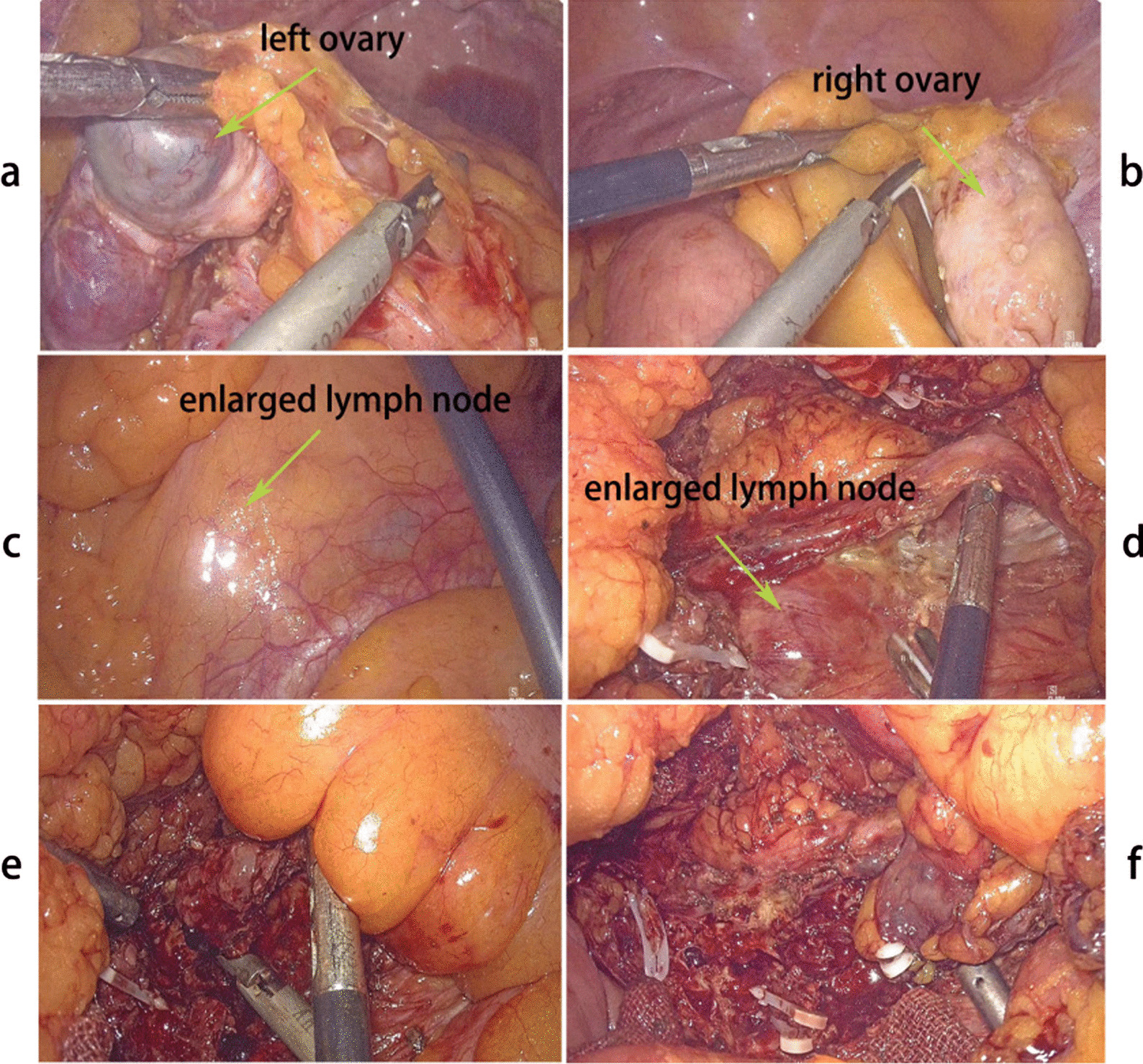
Intraoperative presentation. The shape and size of both ovaries were normal, while the left ovary was cystic-solid (**a**, **b**). An enlarged lymph node-like tissue with moderate hardness about 8 × 4 × 3 cm^3^ in size was found near the abdominal aorta (**c**), which was poorly demarcated from the abdominal aorta and renal artery (**d**). Some lymph node tissue-like samples were excised for intraoperative frozen sections (**e**). The lymph node-like tissue was subsequently removed as much as possible (**f**)

**Fig. 3:**
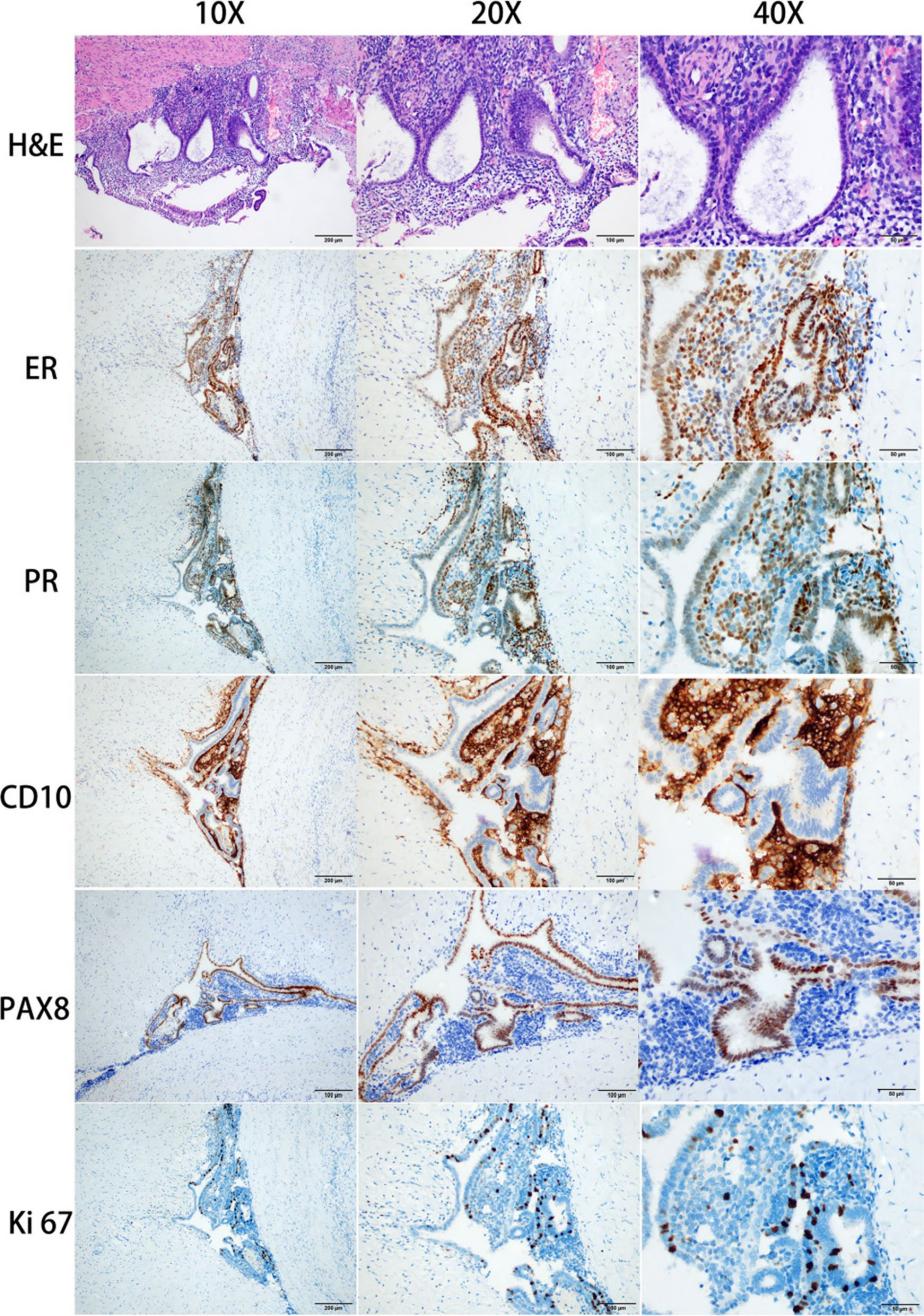
H&E (X10, X20, X40) showed endometrial tissue, stroma and haemosiderin laden macrophages. Immunohistochemistry (X10, X20, X40) showed positive for ER, PR, CD10, PAX 8, Ki 67 10% positive

Laparoscopic surgery was performed 1 week after admission, and no other abnormalities were found during exploration of the abdominal cavity. The shape and size of both ovaries were normal, while the left ovary was cystic-solid (Fig. 2a, b). After opening the retroperitoneal space, an enlarged lymph node-like tissue with moderate solidity and measuring about 8 × 4 × 3 cm^3^ was found near the abdominal aorta (Fig. 2c). When the surrounding adhesions were separated, lymph node-like tissue was poorly demarcated from the abdominal aorta and renal artery (Fig. 2d), with dark red blood clots and interstitial tissue discharging from it. Some lymph node-like tissue samples were sent for intraoperative frozen sections, which demonstrated a benign lesion, with endometrium-like features (Fig. 2e). Because the first intraoperative frozen sections suggested the possibility of a benign lesion that was closely related to the abdominal aorta and renal artery, and considering the great surgical risk of complete resection, the lymph node-like tissue was only excised as much as possible (Fig. 2f). The second set of intraoperative frozen sections were sent, which also suggested a high possibility of endometrial lesions. The final histopathological examination demonstrated the presence of endometriosis in a para-aortic lymph node and left ovary. Immunohistochemistry was positive for estrogen receptor (ER), progesterone receptor (PR), CD10, paired box 8 (PAX 8), and Ki 67 at 10% positive (Fig. 3).

The patient's serum female hormone levels (AMH: < 0.02 ng/ml, E2: < 15 pg/ml, P: 0.20 ng/ml, FSH: 62.08 mIU/ml, LH: 42.07 mIU/ml) were still at postmenopausal levels after surgery, but because of the high preoperative CA125 and suspicion of malignant lesion on imaging, MDT had another discussion, and the diagnosis of endometriosis in a para-aortic lymph node metastasis was made. Gonadotropin-releasing hormone antigen (GnRH-a) treatment every 28 days was recommended.

Presently, the serum CA125 has decreased to normal levels, and no para-aortic lesions were detected on abdominal enhancement CT. She is being followed up regularly.

## Discussion and conclusions

Recent studies have confirmed that EMs are associated with a variety of etiologies, including genetics, epigenetics, metabolomics, immunological stress and inflammatory [6–10]. Several studies about the etiologies of EMs are as follows: (a) Genetics has been found to play a role in the etiology of EMs after long-term familimal studies. In a recently published review, it was proposed that the pathogenesis of EMs may be linked to genes [6], with related genes including metabolism regulation and DNA reparation, inflammation and immune response, steroidogenesis and sex hormone receptorial activity, tissue remodeling and neoangiogenesis, among others; (b) Some researchers have held the view that EMs may be an epigenetic disorder. Evidence has found that EMs has a unique epigenetic expression profile [11]. Furthermore, DNA methylation, histone modifications and microRNAs played a part in modulating the proliferation, invasion and stemness of endometriotic cells [12]; (c) Metabolomics researches may provide serological diagnostic indicators or new biomarkers for EMs. In the study of Murgia F, 1H-NMR analysis of serum samples from patients with and without EMs revealed a decrease in tryptophan, an increase in glutamine and β-hydroxybutyric acid, and alterations in glutamine and glutamate metabolism, pyrimidine metabolism, nitrogen metabolism, and aminyl-trna biosynthetic pathways in patients with EMs [8]; (d) Lipopolysaccharide (LPS) has been considered to be a marker of altered intestinal permeability and microbial translocation substitution. (e) Viganó et al. have detected significantly higher plasma LPS levels in patients with EMs than in healthy groups [9]. The loss of gastrointestinal barrier integrity may contribute to elevated plasma LPS concentrations. Altered small intestinal permeability in patients with EMs leads to enhanced LPS translocation and chronic low-grade inflammation; (f) The immune response appears to play a key role in the pathogenesis of EMs and there is several scientific evidence that the immune response may be modulated by microorganisms. Interestingly, D'alterio's review found a significant increase in Negative Bacteria, Mycobacterium-like and Aspergillus in patients with EMs. The findings have suggested that there may be significant differences in the microbiome profiles of patients with and without EMs [13]; (g) Laganà AS and his colleagues have found a significant increase in the number of macrophages in patients with EMs, which may give rise to an early pro-inflammatory microenvironment and late pro-fibrotic activity [14]. In addition, it has been suggested that leukocytes and peritoneal inflammatory mediators may contribute to the microenvironment in which EMs occur and develop [15].

The pathogenesis of EMs has been well studied, but the mechanism of lymph node metastasis is unknown. The two existing hypotheses include the Müllerian system of secondary chemotaxis and the lymphatic circulation theory of metastasis of endometrial tissue. A recent study has demonstrated that both the potential of lymphangiogenesis and the density of lymph vessels in EMs patients are elevated. Additionally, EMs causes lymph node immune response deficiency and insufficient clearance of endometrial cells, resulting in EMs metastasis [16]. EMs lymph node metastasis was first reported by Li Volsi et al. in 1974, with rare later reports. In 1996, Insabato et al. reported 3 cases of intestinal EMs lymph node metastasis [17]. The exact prevalence of lymph node metastasis in EMs remains unfathomamble because of the lack of epidemiological studies.

This report presents the case of a perimenopausal woman who had an elevated CA125 and enlarged para-aortic lymph node after hysterectomy with bilateral salpingectomy with conservation of both ovaries and was ultimately diagnosed with endometriosis in a para-aortic lymph node. There are three previous reports in the literature (Table 1). In 2011 Beavis et al. reported the first case of EMs of para-aortic lymph nodes of a pregnant woman [18]. The patient was a 25-year-old multipara pregnant woman with a history of chronic pelvic pain and ovarian cystectomies for bilateral endometriomas. At the age of 19 years. During pregnancy, she underwent pelvic MRI because of central placenta previa and subchorionic hemorrhage, which revealed a large complex adnexal mass measuring 10.3 × 8.5 × 5.0 cm with areas of cystic degeneration and possible blood products, as well as 2-cm left obturator lymph. The patient subsequently underwent a lower segment cesarean section at 26^+2^ weeks of gestation because of vaginal bleeding and a lack of adequate response to administration of uterine tocolytics for fetal maturity. The right para-aortic lymph node measured 2.4 cm in its greatest dimension, and microscopy revealed lymphatic tissue composed of endometrial glands surrounded by a cuff of endometrial stroma with decidual changes. Two years later, Escobar et al. [19] reported a case of left adnexal complex mass with an elevated serum CA125 level and an enlarged para-aortic lymph node. Both intraoperative frozen sections and postoperative pathology confirmed left ovarian endometrioma and an enlarged para-aortic lymph node involved with EMs. Recently, Christebull et al. [20] reported a case of primary infertility caused by EMs, with an abnormally elevated CA125 level and an enlarged para-aortic lymph node of about 3 cm diameter. The histopathologic examination revealed endometrial components in a para-aortic lymph node. Since lymph node resection in ovarian and peritoneal EMs is extremely rare, it is unclear whether lymph node metastasis exists in ovarian and peritoneal EMs. In the prospective study of Tempfer et al. [21], 26 patients with suspected ovarian and peritoneal EMs were included, and intraoperative sentinel lymph node staining and dissection were performed. Of the 26 patients, 23 had postoperative histopathological confirmation of EMs. Of the 19 sentinel lymph nodes successfully sampled, 2 (11%) had endometrial cell sentinel lymph node involvement. Immunohistochemistry of the specimens suggested an endometrial stromal origin, with the presence of endometrioid cells in pelvic sentinel lymph nodes.

**Table 1:** Reported cases of endometriosis in para-aortic lymph nodes

References	Publication year	Country	Age, y	Related lesions	Size of node
Beavis et al. [18]	2011	USA	25	Left ovarian mass with obturator lymph node	2.4 cm
Escobar et al. [19]	2013	USA	23	Adnexal mass	Not mentioned
Christable et al. [20]	2020	India	26	Adnexal mass	3 cm
Present case	2021	China	54	None	8 × 4 × 3 cm^3^

Evaluation for the possibility of pelvic endometriosis involves a combination of clinical symptoms, signs imaging studies. However, the gold standard for diagnosing pelvic endometriosis is laparoscopic visualization of the pelvis. There are no specific indicators for preoperative diagnosis of EMs of the para-aortic lymph nodes. MRI has a specific predictive value for EMs, but its accuracy and sensitivity need to be confirmed by more studies. The diagnosis of EMs with lymph node involvement has limitations, and more clinical cases are needed to enrich the physician's experience to improve the accuracy of diagnosis with tissue sample histopathology and immunohistochemical staining. As research progresses and more studies of lymph node involvement in EMs appear in the literature, some scholars have questioned the malignant potential of EMs [22]. Two main relationships have been proposed to describe between EMs and ovarian carcinoma: (1) the coexistence of both as the result of common risk factors and their influence; and (2) the gradual transformation of endometrial cells into tumour cells. Although EMs is not malignant, it shares similarities with cancer, such as cellular infiltration, unrestricted growth, neovascularization, and reduced apoptosis [23]. Ovarian clear cell carcinoma is often associated with EMs. Molecular pathways related to ARID1A mutations have been published, suggesting progression from EMs to subsequent endometriosis-associated ovarian cancer [24]. Before that, one research showed that the expression of ARID1A decreased in vaginal-rectal EMs and involved pelvic sentinel lymph nodes. Suggesting that the decrease might be associated with malignant transformation of EMs [25]. Notably, in another study, 11 (42.3%) of 26 patients with colorectal EMs, had lymph node involvement. Furthermore, lymphovascular infiltration was demonstrated by immunohistochemistry in 4 (36.3%) of 11 patients with lymphatic involvement and in 2 (13.3%) of 15 patients without [26]. Nonetheless, in a study by Rossini et al. [27], 140 patients with deep infiltrating intestinal EMs (One group of 70 patients had EMS lymph node involvement, and another group of 70 patients without) were included, and there were no statistically significant differences in intestinal obstruction, depth of invasion into the intestine, and disease recurrence rates between the two groups; only preoperative plasma CA125 levels were different, leading to the conclusion that lymph node involvement in EMs does not accelerate disease progression. Deep infiltrating EMs are similar to low-grade malignant mesenchymal sarcomas, but unlike them, EMs do not cause death.

The objective of surgery is to remove as much of the endometriosis as possible, and when there is a high risk of lymph node involvement and/or recurrence, removal of only the visible lesions may not be justified. Resection of all visible lesions combined with medication seems less effective than extensive surgery in reducing the risk of recurrence [28]. Hence, further research is needed to determine the best treatment protocol.

It is known that the incidence of lymph node involvement in EMs is relatively rare and that a past medical history may be grounds for suspicion of the diagnosis. There are no established preoperative serum tumor markers of lymph node involvement in extra-pelvic EMS, and imaging studies tend to suggest the presence of a malignancy**.** Histopathology and immunohistochemistry played a crucial role in reaching a diagnosis in this patient. Our report supports the concept that EMs has the potential to invade and metastasize into lymph nodes via the lymphatic vessels.

## Data Availability

All data related to this case report are available from the corresponding author by request.

## Abbreviations

EMs: Endometriosis
CA125: Carbohydrate antigen 125
MRI: Magnetic resonance imaging
PET-CT: Positron emission tomography and computed tomography
AMH: Anti Mullerian hormone
FSH: Follicle-stimulating hormone
LH: Luteinizing hormone
MDT: Multidisciplinary team
ER: Estrogen receptor
PR: Progesterone receptor
